# Evaluation of Preoperative Factors as Predictors of Difficult Thyroidectomy: A Prospective Study

**DOI:** 10.7759/cureus.77982

**Published:** 2025-01-25

**Authors:** Anshuman Sikka, Abhilash Goyal, Kirti Khandelwal

**Affiliations:** 1 General Surgery, BronxCare Health System, New York, USA; 2 General Surgery, Vardhman Mahavir Medical College and Safdarjung Hospital, New Delhi, IND; 3 General and Minimal Access Surgery, All India Institute of Medical Sciences, Guwahati, IND; 4 Head and Neck Surgery, Tata Memorial Hospital, Mumbai, IND; 5 Otorhinolaryngology, Vardhman Mahavir Medical College and Safdarjung Hospital, New Delhi, IND

**Keywords:** difficult thyroidectomy, goiter, recurrent laryngeal nerve injury, thyroid, thyroidectomy, thyroidectomy difficulty scale

## Abstract

Introduction

Thyroidectomy is one of the most common surgical procedures performed worldwide. The complications from thyroidectomy are less, yet they can be life-threatening. The purpose of our study was to evaluate the role of preoperative clinical and biochemical factors in predicting difficult thyroidectomy (DT) and its influence on complications.

Methodology

Fifty-two patients were enrolled in the study, all of whom underwent total or hemithyroidectomy. Preoperative clinical parameters, including large goiter, presence of compressive neck symptoms, retrosternal extension, duration of goiter, and presence of toxic symptoms, were noted. Biochemical tests, including thyroid function tests and thyroglobulin levels, were performed preoperatively. Intraoperative difficulty in thyroidectomy was assessed using the thyroidectomy difficulty scale (TDS). The correlation between complications and TDS was evaluated.

Results

Preoperative clinical features like the presence of compressive neck symptoms (p=0.003), the presence of a large goiter (p=0.0004), the presence of retrosternal extension (p=0.014), and the long duration of goiter (p=0.0003) were significantly associated with DT. Biochemical features like raised serum thyroglobulin levels were significantly associated with DT (p=0.0004). There was a linear relationship between operating times and TDS scores with higher TDS scores associated with longer operating times. Postoperatively, median serum parathyroid hormone (PTH) levels were found to be significantly lower in the DT group at 48 hours postoperatively (p=0.019). Vocal cord palsy was found to be statistically significant (P-value: 0.0013) in the DT group.

Discussion and conclusions

The TDS and longer operating times were used to categorize DTs. Preoperative clinical features can help the surgeon predict whether thyroid surgery will be classified as a DT and alert them to the potential for postoperative complications in this patient group.

## Introduction

Thyroidectomy is one of the most common surgical procedures worldwide [1]. However, during the early part of the 20th century, thyroidectomy was associated with high mortality and morbidity rates, and thyroid surgeries were considered barbaric and banned by the French Medical Society because of the associated mortality. With the advent of aseptic techniques, a better understanding of thyroid anatomy and physiology, improved surgical methods, and the introduction of technological innovations, thyroid surgeries have become much safer. Theodor Kocher achieved a mortality rate of 1% and won the Nobel Prize in 1909 for advancing thyroid surgery [2]. As of today, thyroidectomy is associated with virtually zero mortality and a meager morbidity rate when performed by high-volume surgeons [3-5]. The incidence of perioperative complications is around 1-2% yet complications from thyroidectomy can be life-threatening [6]. Besides, these complications dramatically decrease the quality of life of the patients. Hence, it has always been a topic of keen interest and research for head and neck surgeons. Major complications of thyroidectomy include injury to the recurrent laryngeal nerves (RLNs), causing hoarseness, respiratory distress, and dysphagia; injury to the parathyroid glands, leading to hypocalcemia; and injury to the superior laryngeal nerve, causing aspiration problems [6]. Schneider and colleagues developed the thyroidectomy difficulty scale (TDS), which consists of four items (vascularity, friability, mobility/fibrosis, and gland size) on a 20-point scale, with each item scored on a five-point scale. The minimum score is 4, while the maximum score is 20 [7,8]. The purpose of our study was to identify preoperative clinical and biochemical features that could predict a difficult thyroidectomy (DT) by correlating with the intraoperative TDS score. We also assessed the incidence of postoperative complications in DTs compared to non-difficult cases.

## Materials and methods

The study was a prospective observational study conducted in the Department of Surgery in a tertiary care hospital in New Delhi. The primary objective of our study was to identify the high-risk preoperative clinical features predictive of DT using correlation with intraoperative TDS. The secondary objective was to assess the difference in the incidence of postoperative complications in DTs compared to non-difficult ones. Patients aged above 12 years with benign or malignant thyroid disorders undergoing hemithyroidectomy or total thyroidectomy were included in the study. Patients with neck dissection, renal dysfunction, alcoholic cirrhosis, pregnancy, a history of head and neck cancer within the last five years, prior calcium supplementation, or any other condition that affects calcium metabolism were excluded. Also excluded were those with pre-existing metabolic disorders like secondary hyperparathyroidism, conditions involving parathyroid or bone diseases, pre-existing vocal cord weakness or dysfunction, or a prior history of receiving head and neck radiation.

Fifty-two patients were enrolled in the study, and their clinical history was recorded, along with any co-morbidities. Clinical parameters, including large goiter (>12.5 cm in the vertical axis and >10 cm in the horizontal axis), presence of compressive neck symptoms, retrosternal extension, short neck, duration of goiter ≥ 5 years, and presence of toxic symptoms, were also noted. Biochemical parameters included thyroid function test (hyperthyroid or hypothyroid), serum thyroglobulin levels (Tg), presence of anti-TPO antibodies, serum parathormone levels, and serum calcium level. In the present study, the following reference values were used: serum parathyroid hormone (PTH) 15-65 pg/mL, ionized calcium 4.0-4.8 mg/dL, total calcium 8.5-10.5 mg/dL, thyroglobulin <125 ng/mL, and serum TSH 0.4-6.2 mIU/mL.

Fine needle aspiration cytology was done and reported in Bethesda staging. As a protocol, vocal cord examination was done in all patients preoperatively. It was only after the patient became euthyroid that they were taken for surgery. All the patients were taken up for surgery under general anesthesia with tracheal intubation, and an appropriate type of thyroidectomy was performed. Intraoperative DT scoring encompassed vascularity, friability, mobility/fibrosis, and gland size. TDS score was recorded by the same operating surgeon on a scale of 1-5 assigned to each of the four items as mentioned above. Operative times were obtained from the electronic medical record and calculated as the time from incision until wound closure. Surgical time for hemithyroidectomy was doubled compared to total thyroidectomy. A TDS score of 12 or above and duration of surgery ≥120% of the mean operating time was categorized as DT. Parathyroid glands and the RLN (bilateral in total thyroidectomy and unilateral in hemithyroidectomy) were identified in all cases and well preserved.

Hypocalcemia was defined as a serum total calcium level less than 8.5 mg/dL or a serum ionized calcium level less than 4 mg/dL, and/or symptoms such as numbness, tingling, tetany, Trousseau's sign, and Chvostek's sign. Persistence of hypocalcemia for more than six months was labeled as permanent hypocalcemia. Hypoparathyroidism was defined as serum PTH levels less than 15 pg/mL. The persistence of hypoparathyroidism for more than six months was labeled as permanent hypoparathyroidism.

Postoperatively all patients were assessed for objective signs and symptoms of hypocalcemia at 48 hours, one month, and six months. The symptoms of hypocalcemia, including paresthesias, numbness, tingling, nausea, tetany, Trousseau's sign, and Chvostek's sign, were evaluated. The patients with symptomatic hypocalcemia were treated with intravenous calcium gluconate injection in addition to oral supplementation. Serum ionized calcium levels and serum PTH levels were evaluated at 48 hours, one month, and six months post-operatively. Hypocalcemia/hypoparathyroidism persisting beyond a period of six months was considered permanent hypocalcemia/hypoparathyroidism. All patients who underwent total thyroidectomy had a detailed thyroid function test (TSH, T4, T3) postoperatively. Vocal cords were assessed at the time of extubation by the anesthetist, as well as one week, one month, and six months after surgery, to check for both transient and permanent vocal cord palsy. Palsy persisting beyond six months was labeled as permanent nerve palsy/vocal cord palsy.

## Results

The study comprised of patients aged between 16 years and 66 years. The mean age was 39.96±13.06 years and the median age was 40 years. Nineteen patients were categorized in the DT group, while 33 patients were in the non-difficult thyroidectomy (NDT) group. The mean age in the DT group was 48.53±11.22 years and in the NDT group was 35.03±11.51 years. A total of 10 patients were aged >55 years, of which eight were in the DT group. Age greater than 55 years was significantly associated with a DT (p-value 0.011). Out of 52 patients, six were male and 46 were female. Our study did not reveal any association of gender with difficulty in thyroidectomy. The number of DTs was significantly higher in the total thyroidectomy group compared to the hemithyroidectomy group as shown in Table 1 (p=0.001).

**Table 1:** Association of type of surgery with difficulty (DT/NDT) DT, difficult thyroidectomy; NDT, non-difficult thyroidectomy

Type of surgery	Procedure		Total	P-value
	DT	NDT		
Total thyroidectomy	15 (53.57%)	13 (46.43%)	28 (100.00%)	0.009
Hemithyroidectomy	4 (16.67%)	20 (83.33%)	24 (100.00%)	
Total	19	33	52	

Out of 52 study subjects, nine were diagnosed as toxic multinodular goiter (MNG), 15 as non-toxic MNG, and 28 as solitary thyroid nodules (STN). All toxic MNG patients were found to be in the DT group (p-value 0.0047) whereas the incidence of DT was significantly low in the patients with STN (p-value <0.0001) as depicted in Table 2.

**Table 2:** Association between preoperative diagnosis and difficulty (DT/NDT) DT, difficult thyroidectomy; NDT, non-difficult thyroidectomy; MNG, multinodular goiter; STN, solitary thyroid nodule

Preoperative diagnosis	Procedure		Total
	DT	NDT	
MNG	9 (60%)	6 (40%)	15 (100.00%)
STN	3 (10.71%)	25 (89.28%)	28 (100.00%)
Toxic MNG	9 (100.00%)	0 (0.00%)	9 (100.00%)
Total	19 (36.54%)	33 (63.46%)	52 (100.00%)

On comparing preoperative thyroid profiles, we found that 71.15% of patients were euthyroid, followed by hyperthyroid (19.23%) and hypothyroid (9.62%). Of the hyperthyroid patients, 80% were in the DT group, while 75.68% of euthyroid patients were in the NDT group, as shown in Table 3. Primary euthyroid status was significantly associated with NDT (p=0.0018).

**Table 3:** Preoperative thyroid status distribution in DT and NDT groups DT, difficult thyroidectomy; NDT, non-difficult thyroidectomy

Preoperative thyroid status	DT	NDT	P-value
Euthyroid	24.32%	75.68%	0.0018
Hyperthyroid	80%	20%	
Hypothyroid	40%	60%	

A total of 10 patients (19.23%) complained of compressive neck symptoms because of thyroid swelling. Eight (80.00%) of these were from the DT group. Clinically retrosternal extension was seen in four patients (7.69%). All four patients were from the DT group. A total of seven patients (13.46%) had large goiter (>12.5 cm in the vertical axis and >10 cm in the horizontal axis) on clinical examination. All seven patients were in the DT group. Duration of goiter varied from one month to 28 years. The mean duration of goiter was calculated to be 4.64 years. A total of 17 patients (32.69%) had a goiter duration of 5 years or more. Out of these, 12 (70.59%) were in the DT group. Hence, the presence of compressive neck symptoms (p=0.003), the presence of a large goiter (p=0.0004), the presence of retrosternal extension (p=0.014), and long duration of goiter (p=0.0003) were significantly associated with a DT as shown in Table 4.

**Table 4:** Association between clinical features, postoperative cord palsy, and difficulty (DT/NDT) DT, difficult thyroidectomy; NDT, non-difficult thyroidectomy

Clinical feature		Procedure		Total	P-value
		DT	NDT		
Compressive neck symptoms	Present	8 (80.00%)	2 (20.00%)	10	0.003
	Absent	11 (26.19%)	31 (73.81%)	42	
Large goiter	Present	7 (100.00%)	0 (0.00%)	7	0.0004
	Absent	12 (26.67%)	33 (73.33%)	45	
Duration of goiter (years)	≥5	12 (70.59%)	5 (29.41%)	17	0.0003
	<5	7 (20.00%)	28 (80.00%)	35	
Retrosternal extension on clinical examination	Present	4 (100%)	0 (0.00%)	4	0.014
	Absent	15 (31.25%)	33 (68.75%)	48	
Postoperative vocal cord palsy	Transient	5 (100%)	0	5	0.0013
	Permanent	1 (100%)	0	1	
	Absent	13 (28.26%)	33 (71.74%)	46	

Additionally, the sensitivity, specificity, PPV, and NPV of the above-mentioned clinical factors in predicting DT are depicted in Table 5.

**Table 5:** Sensitivity, specificity, PPV, and NPV of clinical parameters for predicting a DT PPV, positive predictive value; NPV, negative predictive value; DT, difficult thyroidectomy

Clinical parameters	Sensitivity	Specificity	PPV	NPV
Presence of compressive neck symptoms	42.11%	93.94%	80.00%	73.81%
Large goiter	36.84%	100.00%	100.00%	73.33%
Presence of retrosternal extension on clinical examination	21.05%	100.00%	100.00%	68.75%
Duration of goiter ≥5 years	63.16%	84.85%	70.59%	80.00%

Raised serum TSH levels were not significantly associated with DT (p=1.00). Serum thyroglobulin levels ≥125 ng/mL were seen in seven patients (13.46%). All seven patients were from the DT group.

**Figure 1:**
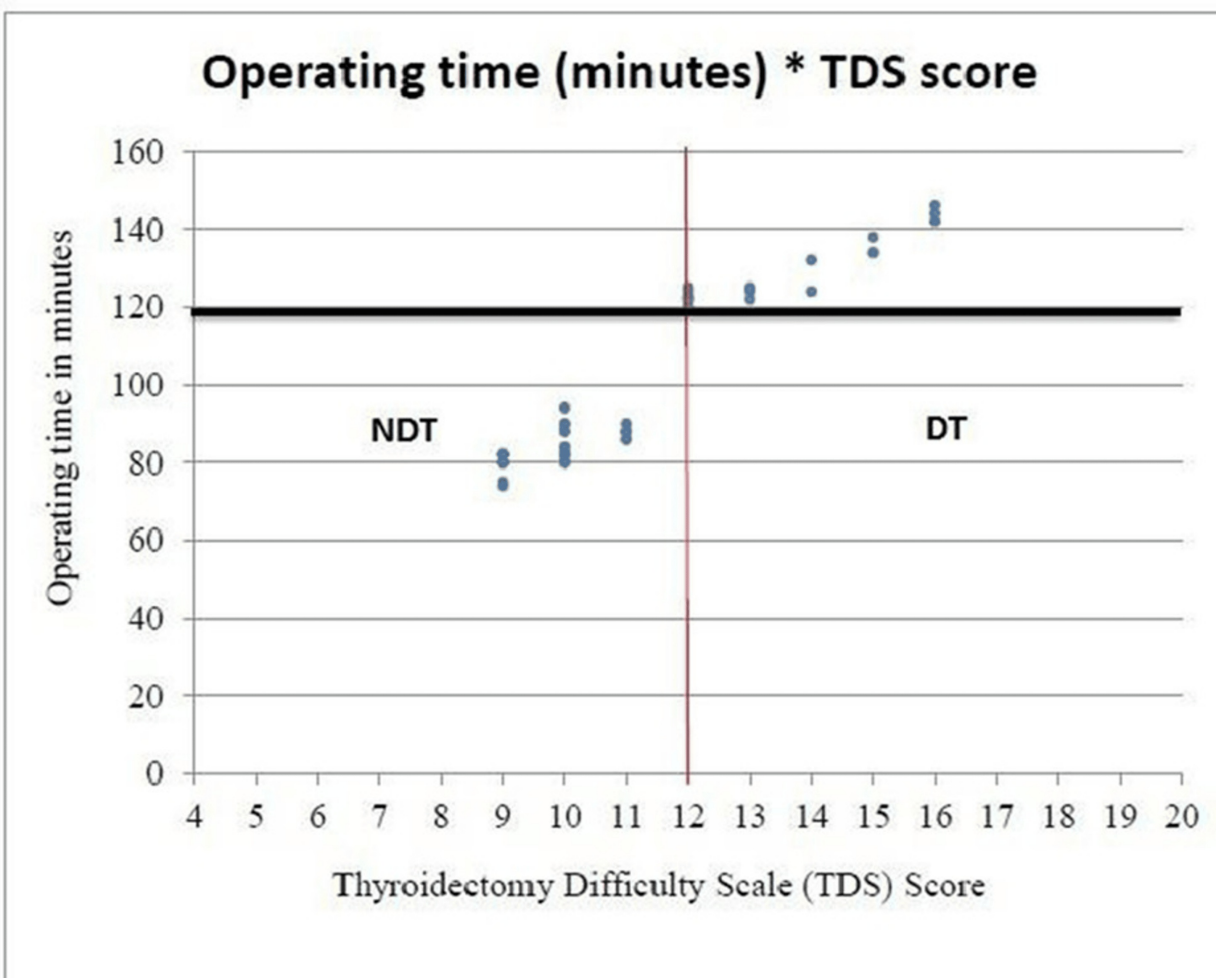
Distribution of cases on the basis of TDS scores and operating time (minutes) The red line shows a cut-off TDS of 12. The black line represents 120% of the mean operating time of 119.82 minutes (or 1.997 hours). 120 minutes corresponds to the absolute cut-off value for an operating time of two hours. DT, difficult thyroidectomy; NDT, non-difficult thyroidectomy; TDS, thyroidectomy difficulty scale

Raised serum thyroglobulin levels were significantly associated with DT (p=0.0004). Serum anti-TPO antibodies were present in 15 patients (28.85%). Out of these, six (40%) were in the DT group and nine (60%) were in the NDT group. The presence of serum anti-TPO antibodies was not significantly associated with DT (p=0.741). On univariate regression analysis, age >55 years, duration of goiter ≥5 years, presence of large goiter, compressive neck symptoms, toxic MNG, MNG, hyperthyroidism, total thyroidectomy, and raised serum thyroglobulin (Tg) levels were significantly associated with a DT. The mean operating time was 99.85±22.88 minutes or 1.66 hours. There was a linear relationship between operating times and TDS scores with higher TDS scores associated with longer operating times. Spearman's coefficient of rank correlation ρ (rho) was 0.922 and R2 was 0.8501. The correlation between TDS scores and operating times was statistically significant (p<0.0001) as shown in Figure 1.

**Figure 2:**
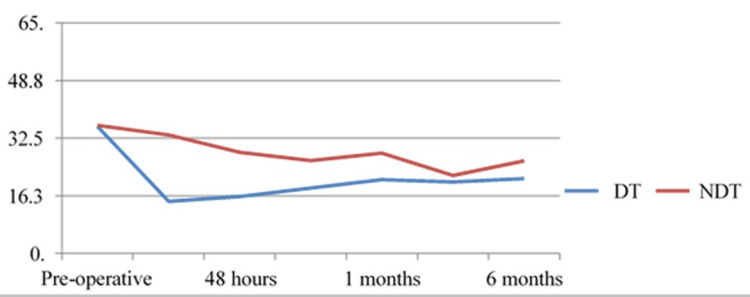
Median serum PTH levels in DT and NDT groups DT, difficult thyroidectomy; NDT, non-difficult thyroidectomy; PTH, parathyroid hormone

Postoperatively, patients were assessed on the basis of serum total calcium, ionized calcium, serum PTH, and simultaneous clinical evaluation for symptoms of hypocalcemia. None of the patients who underwent hemithyroidectomy developed postoperative hypocalcemia or hypoparathyroidism. Ten out of 28 patients undergoing a total thyroidectomy showed hypocalcemia postoperatively, out of which nine (90%) were of the DT group. Only one patient had persistent hypocalcemia at six months postoperatively. The mean serum ionized calcium levels were lower in the DT group compared to the NDT group at all postoperative intervals of measurement. However, the difference in the two categories was not statistically significant. Ten patients out of 28 patients developed hypoparathyroidism at 48 hours postoperatively, out of which eight (80%) were of the DT group. Only one patient remained hypoparathyroid at the end of six months postoperatively. The median serum PTH levels were found to be significantly lower in the DT group at 48 hours postoperatively (p=0.019) as shown in Figure 2.

Vocal cords were assessed at the time of extubation as well as after one week, one month, and six months of surgery to look for transient as well as permanent vocal cord palsy. A total of six patients had vocal cord palsy after one week of surgery. All six patients belonged to the DT group, and the relation was found to be statistically significant (P-value 0.0013). Of these, only one patient continued to have palsy at six months after surgery as shown in Table 4. In all the cases, it was unilateral vocal cord palsy.

## Discussion

Surgical procedures have the potential for complications, and thyroidectomy is no exception. Surgery of the thyroid takes place in an area of complicated anatomy. In the present study comprising 52 patients, the mean age was 39.96±13.06 years and 88.46% of the patients were female. This distribution was similar to a study by Zambudio AR et al. [9], where the mean age was 48±14 years, and 89% of the patients were female. Mok VM et al. [8] studied 146 patients, where 77.25% of patients were female and the mean age was 49±15.0 years. In the present study, 65.38% of patients were asymptomatic while the most common clinical features in the rest of the patients were compressive neck symptoms (19.23%) and hyperthyroidism (19.23%). Zambudio AR et al. [10] reported compressive symptoms in 29% of patients. Lacoste et al. [10] in a series of over 3000 thyroidectomies found that 11% of patients complained of compression symptoms. The difference in the incidence of compressive symptoms can be attributed to the variation in the number of cases and the distribution of patients with large goiters, thyroiditis, and carcinoma, all of which can cause compressive symptoms. In the present study, 71.25% of patients were euthyroid while 19.23% of patients had hyperthyroidism followed by hypothyroidism in 9.61% of patients. Sarma MK et al. [11] found 81.2% of cases to be in euthyroid status preoperatively, followed by 12.2% cases in hypothyroid and 6.4% cases in hyperthyroid status. The most common presentation in our study was STN in 28 patients (53.85 %) followed by MNG in 24 (46.15 %) cases (nine being toxic MNG). Gupta S et al. [12] also had a similar finding. MNG (38.2%) and STN (30%) were also the most common indications for thyroid surgery as reported by Zakaria HM et al. [13]. The most common pathology observed in the thyroid gland needing surgical intervention was colloid goiter (79.59%). This was similar regarding the distribution of type of thyroid surgery and FNAC report findings in studies done by Sarma MK et al. [11], Gupta S et al. [12], and Zambudio et al. [9]. In the present study, raised serum thyroglobulin levels were significantly associated with DT (p=0.0004). Mok et al. [8] reported that the presence of anti-thyroglobulin antibodies (p=0.015) and high thyroglobulin (p=0.037) was independently associated with DT.

In the present study, a TDS score of 12 or more was labeled as DT. In addition to this scale, a surgery duration ≥120% of the mean operating time (1.66 hours) was classified as DT. In our study, 19 procedures (36.54%) were DT and the number of total thyroidectomies was significantly higher in the DT group (p=0.001). The mean TDS score was higher in the DT group. Mok VM et al. [8] and Schnieder DF et al. [7] also observed higher TDS scores for the DT group, which correlated with operating times. In the present study also, on comparing the operating times of the DT group to the NDT group, there was a linear relationship between operating times and TDS scores (higher TDS scores associated with longer operating times).

Transient hypocalcemia was seen in a total of nine patients while permanent hypocalcemia was seen in one patient. Similarly, nine patients had transient hypoparathyroidism and one remained hypoparathyroid at six months. None of the patients undergoing hemithyroidectomy had postoperative symptomatic hypocalcemia. Observational studies by Nair CG et al. [14] have noted up to 50% of transient and 4% permanent hypocalcemia after thyroidectomy. Schneider DF et al. [7] reported temporary hypocalcemia in 60.9% of patients. Zambudio et al. [9] observed transient hypoparathyroidism/hypocalcemia in 9.63% of cases and permanent hypoparathyroidism/hypocalcemia was observed in 0.66% of cases. The lower rates reported by Zambudio et al. [10] compared to our study can be attributed to the higher number of patients in their study and a lower cut-off value for serum calcium of 7.5 mg/dL (compared to 8.5 mg/dL in our study) in asymptomatic cases. Ritter K et al. [15], in a study of 1,054 patients, observed 18% of patients with transient hypoparathyroidism and 1.9% of patients with permanent hypoparathyroidism. The difference in the incidence rates can be explained on the basis of the difference in the number of patients in their study compared to our study and the lower cut-off value of serum PTH level <10 pg/mL to label hypoparathyroidism (<15 pg/mL in the present study). RLN injury is a common severe complication in thyroid surgery and unilateral RLN injury is more common. Transient RLN palsy affects 5-10% of patients after extracapsular thyroidectomy [16]. In the present study, a total of six patients (11.54 %) had unilateral vocal cord palsy after one week of surgery. Of these, only one patient (1.92%) also had vocal cord palsy at six months after surgery. Thus a total of five patients (9.62%) had transient vocal cord palsy while one patient (1.92%) had permanent vocal cord palsy. In a study done in China by Jiang Y et al. [17], the incidence of RLN injury was 4.98%. In another study done in Saudi Arabia by Zakaria HM et al. [13], the incidence of RLN injury was 4.1%. Identification of the RLN during surgery minimizes the risk of injury. In our study, the RLN was identified in all the surgeries and we recommend the same. Total/near-total thyroidectomy was significantly associated with a greater risk of RLN injury compared to other types of surgery in a study by Zakaria HM et al. (p=0.024) [13]. In our study, all the patients with vocal cord palsy (temporary as well as permanent) were from the DT group and were not significantly associated with the type of surgery. In a study by Sarma MK et al. [11], all the vocal cord palsies that occurred were observed post-hemithyroidectomy. Thus, rather than the type of surgery, it may be the intraoperative "difficulty" that places the RLN at higher risk during surgery. These are situations where adjuncts, such as intraoperative nerve monitoring for the RLN [18] and indocyanine green for parathyroid identification [19], might help reduce complications.

The limitations of the study include its single-center design and limited sample size. However, a strength of the study is that it was prospective and conducted with clear, specific objectives. Additionally, the surgery was performed by a single chief surgeon, which reduced inter-surgeon variability.

DT was significantly associated with increased age (age >55 years), duration of goiter of five or more years, toxic MNG, presence of retrosternal extension, presence of large goiter, total thyroidectomy, compressive neck symptoms, mean serum PTH levels at 48 hours, and transient vocal cord palsy. The TDS incorporates all of these aspects of difficulty - size, vascularity, friability, and gland size - to create a single composite score of difficulty. Not only is the TDS score correlated with operative time, but it can also improve our understanding of the specific factors contributing to difficulty.

## Conclusions

Clinical preoperative factors such as advanced age (>55 years), the presence of compressive neck symptoms, a large thyroid size, and a history of swelling lasting more than five years were significantly associated with DT. Postoperative complications, including hypocalcemia, hypoparathyroidism, and vocal cord paralysis, occurred more frequently in the DT group. Identifying high-risk preoperative features can help surgeons predict DTs. The use of the intraoperative TDS scale can prompt surgeons to exercise greater caution during the perioperative period. The use of adjuncts for identifying the parathyroid glands and RLN can be explored in DTs. 
